# Identification of GRB2 and GAB1 Coexpression as an Unfavorable Prognostic Factor for Hepatocellular Carcinoma by a Combination of Expression Profile and Network Analysis

**DOI:** 10.1371/journal.pone.0085170

**Published:** 2013-12-31

**Authors:** Yanqiong Zhang, Zhiwei Li, Mei Yang, Danhua Wang, Lingxiang Yu, Chaonan Guo, Xiaodong Guo, Na Lin

**Affiliations:** 1 Research center of traditional Chinese medicine theory, Institute of Chinese Materia Medica, China Academy of Chinese Medical Sciences, Beijing, China; 2 Department of Pathology and Hepatology, Beijing 302 Hospital, Beijing, China; Yonsei University College of Medicine, Korea, Republic Of

## Abstract

**Aim:**

To screen novel markers for hepatocellular carcinoma (HCC) by a combination of expression profile, interaction network analysis and clinical validation.

**Methods:**

HCC significant molecules which are differentially expressed or had genetic variations in HCC tissues were obtained from five existing HCC related databases (OncoDB.HCC, HCC.net, dbHCCvar, EHCO and Liverome). Then, the protein-protein interaction (PPI) network of these molecules was constructed. Three topological features of the network ('Degree', 'Betweenness', and 'Closeness') and the k-core algorithm were used to screen candidate HCC markers which play crucial roles in tumorigenesis of HCC. Furthermore, the clinical significance of two candidate HCC markers growth factor receptor-bound 2 (GRB2) and GRB2-associated-binding protein 1 (GAB1) was validated.

**Results:**

In total, 6179 HCC significant genes and 977 HCC significant proteins were collected from existing HCC related databases. After network analysis, 331 candidate HCC markers were identified. Especially, GAB1 has the highest k-coreness suggesting its central localization in HCC related network, and the interaction between GRB2 and GAB1 has the largest edge-betweenness implying it may be biologically important to the function of HCC related network. As the results of clinical validation, the expression levels of both GRB2 and GAB1 proteins were significantly higher in HCC tissues than those in their adjacent nonneoplastic tissues. More importantly, the combined GRB2 and GAB1 protein expression was significantly associated with aggressive tumor progression and poor prognosis in patients with HCC.

**Conclusion:**

This study provided an integrative analysis by combining expression profile and interaction network analysis to identify a list of biologically significant HCC related markers and pathways. Further experimental validation indicated that the aberrant expression of GRB2 and GAB1 proteins may be strongly related to tumor progression and prognosis in patients with HCC. The overexpression of GRB2 in combination with upregulation of GAB1 may be an unfavorable prognostic factor for HCC.

## Introduction

Hepatocellular carcinoma (HCC) accounts for one of the most common malignant tumors and the third leading cause of cancer-related deaths worldwide [1]. The distribution of HCC is unbalanced throughout the world, with the highest incidence in Asia and Sub-Saharan Africa, especially in China, an endemic area with almost one third of the HBsAg carriers worldwide. The overall 5-year survival rate for HCC patients is still only 5% [2]. Approximately 70% of patients may relapse within 5 years after surgery and more than 80% of postoperative recurrence occurs in the remnant liver [3,4]. Several attempts have been made to predict the occurrence and prognosis of HCC based on single or multiple clinicopathologic features such as the severity of the liver function, age, tumor size, grade, microvascular invasion, portal vein thrombosis, and the presence of microsatellite regions [5,6]. However, HCC patients with the same clinicopathologic features often display different outcome, suggesting that there may be several complex molecular and cellular events involved in the development and aggressive progression of HCC. Thus, elucidating the molecular mechanisms underlying tumor progression and identifying the key markers that differentiate the occurrence and the various stages of HCC are essential for developing novel prognostic factors and improve therapeutic strategies.

With the development of high-throughput methods (such as large-scale genome-wide microarray and mass spectrometry), a wealth of information on biologically relevant systems of human cancer are now available. For example, Lim et al. [7] constructed a molecular prognostic model to predict the disease-free survival in patients with HCC by gene expression profiling; Wang et al. [8] found the common and different characteristics of the three types of liver cancer: HCC, cholangiocarcinoma (CC), and combined HCC-CC (CHC) by comparing their gene expression profilings; Marshall et al. [9] investigated global gene expression profiles from HCC arising in different liver diseases to test whether HCC development is driven by expression of common or different genes, which could provide new diagnostic markers or therapeutic targets. However, accumulating studies have found that crucial disease genes and proteins often show relatively slight changes in their expression patterns between normal and disease states, suggesting that the differential expression analysis may miss some slightly differentially expressed but functionally important genes and proteins. Therefore, it is necessary to develop an efficient method to analyze the high-throughput expression profile data in order to uncover important biological relationships.

Since protein-protein interaction (PPI) networks constitute the basis of most life processes, such studies might enable us to systematically realize the behaviors and properties of biological molecules. Rapid advances in network biology indicate that cancer genes and proteins do not function in isolation; instead, they work in interconnected pathways and molecular networks at multiple levels [10]. Our study group has recently developed two systems biology-based classifiers for early diagnosis of HCC and prostate cancer (PCa), respectively, by combining differential gene and protein expression and topological characteristics of human protein interaction networks, and also demonstrated that these classifiers may efficiently enhance the diagnostic performance for HCC and PCa [11,12]. On this basis, in the current study, we intend to collect full-scale HCC related data including HCC significant genes and proteins which were differentially expressed or had genetic variations in HCC tissues relative to their corresponding normal tissues from five existing HCC related databases (OncoDB.HCC [13], HCC.net [14], dbHCCvar [15], EHCO [16] and Liverome [17]). In order to investigate the functional relationships between these significant molecules, we constructed the protein-protein interaction network and analyzed the topological features of nodes to screen novel markers for HCC. We further perform the experimental validation on the clinical significance of candidate HCC markers by immunohistochemistry analysis.

## Materials and Methods

**Figure 1:**
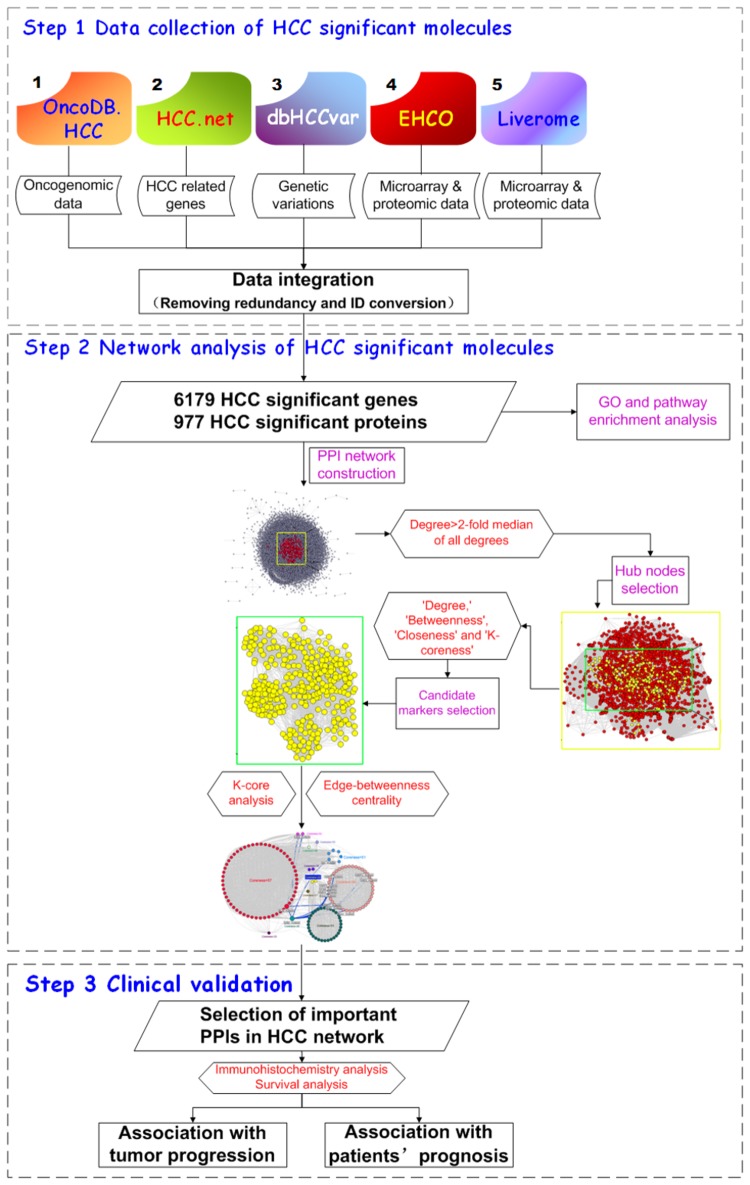
A schematic diagram of this systems biology-based analysis for HCC marker identification. First, HCC significant molecules which were differentially expressed or had genetic variations in HCC tissues relative to their corresponding normal tissues were collected from five existing HCC related databases (OncoDB.HCC, HCC.net, dbHCCvar, EHCO and Liverome). Second, their protein-protein interaction networks were constructed and the hub proteins were chosen according to node degree. Third, a list of candidate HCC markers were identified by calculating four topological features of the network ('Degree', 'Betweenness', ‘Closeness’ and 'K-coreness' ). After that, the key PPIs of candidate HCC markers were selected by K-core analysis and edge-betweenness algorithm. The K-core of a graph is defined as the largest subgraph where every node has at least k links. For each choice of k, we determine the k-cores by iteratively pruning all nodes with degree lower than k and their incident links. The edge-betweenness algorithm is a top-down, divisive method for grouping network components into modules. Edge-betweenness centrality is the frequency of an edge that places on the shortest paths between all pairs of vertices. The edges with highest betweenness values are most likely to lie between sub-graphs. Here, we chose PPI (between RL30_HUMAN and GRB2_HUMAN) with highest edge-betweenness as the most important PPI in HCC network because it may connect different cores with the shortest paths. Finally, the clinical significance of RL30_HUMAN (RPL30) and GRB2_HUMAN (GRB2) was validated using a large cohort of patients with HCC.

The technical strategy of this study was shown in Figure 1.

### Data preparation

#### HCC-significant gene and protein collection

HCC-significant genes and proteins were collected from five HCC related databases, including OncoDB.HCC (Last modified on Apr. 2008, http://oncodb.hcc.ibms.sinica.edu.tw/index.htm), HCC.net (Update time: Apr 18, 2010, http://www.megabionet.org/hcc/index.php), dbHCCvar (Last modified on Sep 4, 2012, http://genetmed.fudan.edu.cn/dbHCCvar/), EHCO (Encyclopedia of Hepatocellular Carcinoma genes Online, Last modified on Sep, 2012, http://ehco.iis.sinica.edu.tw), and Liverome (Last modified on Apr 14, 2011, http://liverome.kobic.re.kr/). For HCC-related molecular events, OncoDB.HCC is the first comprehensive HCC oncogenomic database and contains 577 HCC-related genes [13]. HCC.Net, which has compiled a network with thousands of HCC-related genes identified in different types and stages of HCC, contains 2,131 HCC-related genes [14]. dbHCCvar is an online database which contains 636 human genetic variations and 195 human genes which have been statistically tested to be associated with HCC [15]. EHCO is derived from thirteen gene sets related to HCC and gives 3833 HCC-significant genes [16]. Liverome is a curated database of liver cancer-related gene signatures [17]. There are 6024 gene signatures obtained mostly from published microarray and proteomic studies, and thoroughly curated by experts. In order to facilitate data analysis, the different ID types for HCC significant genes and proteins were converted to ID from UniProtKB-Swiss-Prot/TrEmbL. The detailed information on these HCC significant genes and proteins is described in Table S1.

#### Protein-protein interaction (PPI) data

PPI data were imported from eight existing PPI databases including Human Annotated and Predicted Protein Interaction Database (HAPPI) [18], Reactome [19], Online Predicted Human Interaction Database (OPHID) [20], InAct [21], Human Protein Reference Database (HPRD) [22], Molecular interaction Database (MINT) [23], Database of Interacting Proteins (DIP) [24], and PDZBase [25]. The detailed information on these PPI databases is described in Table S2.

### Gene Ontology (GO) and pathway enrichment analysis for HCC significant genes and proteins

We used Database for Annotation, Visualization and Integrated Discovery [26] (DAVID, http://david.abcc.ncifcrf.gov/home.jsp,version 6.7) for GO enrichment analysis. DAVID now provides a comprehensive set of functional annotation tools for investigators to understand biological meaning behind large list of genes. We also performed pathway enrichment analysis using pathway data obtained from the FTP service of KEGG [27] (Kyoto Encyclopedia of Genes and Genomes, http://www.genome.jp/kegg/, Last updated: Oct 16, 2012). The KEGG PATHWAY section is a collection of manually constructed pathway maps representing information on molecular interaction and reaction networks.

### Network analysis

#### Network construction

HCC significant proteins were used to construct HCC related network. The PPI data were obtained from eight existing PPI databases as mentioned above. Then, we applied Navigator software (Version 2.2.1) and Cytoscape (Version 2.8.1) to visualize the networks.

#### Defining network topological feature set

For each node i in HCC related network, we defined five measures for assessing its topological property: (1) 'Degree' is defined as the number of links to node i; (2) 'Node Betweenness' is defined as the number of edges running through node i. (3) ‘Closeness’ is defined as the inverse of the farness which is the sum of node i distances to all other nodes. The Closeness centrality can be regarded as a measure of how long it will take to spread information from node i to all other nodes sequentially. Degree, node betweenness and closeness centralities can measure a protein’s topological importance in the network. The larger a protein’s degree/betweenness/closeness centrality is, the more important the protein is in the PPI network [28]. (4) K-core analysis is an iterative process in which the nodes are removed from the networks in order of least-connected [29]. The core of maximum order is defined as the main core or the highest k-core of the network. A k-core sub-network of the original network can be generated by recursively deleting vertices from the network whose degree is less than k. This results in a series of sub-networks that gradually reveal the globally central region of the original network. On this basis, 'K value' is used to measure the centrality of node i. (5) 'Edge Betweenness' is defined as the frequency of an edge that places on the shortest paths between all pairs of vertices in network [30]. The edges with highest betweenness values are most likely to lie between sub-graphs.

### Experimental validation

After the expression profile and interaction network analyses, GRB2 and GAB1 were chosen to perform experimental validation in order to investigate the clinical significance of the two proteins in human HCC.

#### Ethics Statement

The study was approved by the Research Ethics Committee of 302nd Hospital of PLA, Beijing, China. Written informed consent was obtained from all of the patients. All specimens were handled and made anonymous according to the ethical and legal standards.

#### Patients and Tissue Samples

A total of 130 patients with primary HCC who underwent a curative liver resection at the 302nd Hospital of PLA, Beijing, China, were included in this study. One hundred and thirty self pairs of HCC and adjacent nonneoplastic liver tissues (as control tissues) obtained from these patients with HCC were retrieved from the tissue bank of the Department of Pathology in the 302nd Hospital of PLA. These patients were diagnosed as HCC between 2001 and 2006. None of the patients recruited in this study had chemotherapy or radiotherapy before the surgery. HCC diagnosis was based on World Health Organization (WHO) criteria. Tumor differentiation was defined according to the Edmondson grading system. Liver function was assessed using the Child-Pugh scoring system. Tumor staging was determined according to the sixth edition of the tumor-node-metastasis (TNM) classification of the International Union against Cancer. The clinicopathological features of 130 patients are summarized in Table 1.

The median follow-up period was 8.6 years. Postoperative surveillance included routine clinical and laboratory examinations every third month, computed tomography scans of the abdomen, and radiographs of the chest every third month. After 5 years, the examination interval was extended to 12 months. 

**Table 1:** Association of GRB2 and GAB1 expression with clinicopathological features of 130 hepatocellular carcinoma patients.

**Clinicopathological Features**	**Case**	**GRB2-high (n, %)**	**P**	**GAB1-high (n, %)**	**P**	**GRB2-high/ GAB1-high (n, %)**	**P**
**Age (years)**							
≤50	72	36 (50.00)	NS	36 (50.00)	NS	30 (41.67)	NS
>50	58	29 (50.00)		31 (53.45)		26 (44.83)	
**Gender**							
Male	96	48 (52.08)	NS	50 (52.08)	NS	42 (43.75)	NS
Female	34	17 (50.00)		17 (50.00)		14 (41.18)	
**Serum AFP**							
Positive	72	47 (65.28)	0.006	41 (56.94)	NS	40 (55.56)	0.01
Negative	58	18 (31.03)		26 (44.83)		16 (27.59)	
**Tumor stage**							
T1	23	0 (0)	0.01	2 (8.70)	0.01	0 (0)	0.006
T2	40	19 (47.50)		21 (52.50)		15 (37.50)	
T3	52	34 (65.38)		33 (63.46)		31 (59.62)	
T4	15	12 (80.00)		11 (73.33)		10 (100.00)	
**Tumor grade**							
G1	31	13 (41.94)	NS	10 (32.26)	0.02	10 (32.26)	0.02
G2	76	37 (48.68)		40 (52.63)		31 (40.79)	
G3	23	15 (65.22)		17 (73.91)		15 (65.22)	
**Growth pattern**							
Trabecular	101	50 (49.50)	NS	50 (49.50)	NS	41 (40.59)	NS
Nontrabecular	29	15 (51.72)		18 (62.07)		15 (51.72)	
**Cirrhosis**							
Yes	86	43 (50.00)	NS	43 (50.00)	NS	38 (44.19)	NS
No	44	22 (50.00)		24 (54.55)		18 (40.91)	
**Underlying liver disease**							
Alcoholic	25	12 (48.00)	NS	14 (56.00)	NS	11 (44.00)	NS
Hepatitis B	49	26 (53.06)		26 (53.06)		22 (44.90)	
Hepatitis C	35	18 (51.43)		18 (51.43)		16 (45.71)	
Unknown	21	9 (42.86)		9 (42.86)		7 (33.33)	

Note: ’NS’ refers to the differences among groups have no statistical significance.

#### Immunohistochemistry analysis

GRB2, GAB1 and phosphorylated ERK1 (p-ERK1) expression were immunohistochemically evaluated in paraffin-embedded specimens of 130 pairs of HCC and adjacent nonneoplastic liver tissues. Surgical specimens were fixed in 10% formalin, embedded in paraffin, and sectioned at a 4 μm thickness. For heat-induced epitope retrieval, deparaffinized sections were soaked in 10 mM citrate buffer (pH 6.0) and treated at 95°C for 30 min using the microwave oven method. Immunohistochemical staining was performed using the avidin-biotin immunoperoxidase technique according to our previous studies [31-33]. The activity of endogenous peroxidase was blocked by incubation with 0.3% H_2_O_2_ in methanol for 15 min, and nonspecific immunoglobulin binding was blocked by incubation with 10% normal goat serum for 10 min. Sections were incubated at room temperature for 4 h with anti-GRB2 rabbit polyclonal antibody (#ab111031, Abcam, Cambridge, United Kingdom), or anti-GAB1 rabbit polyclonal antibody (#ab59362, Abcam, Cambridge, United Kingdom), or anti-phosphorylated ERK1 (phospho Y204) rabbit polyclonal antibody (#ab131438, Abcam, Cambridge, United Kingdom), and were then rinsed and incubated for 30 min with a biotinylated second antibody. After washing, the sections were incubated for 30 min with horseradish peroxidase-conjugated streptavidin, and were finally treated with 3,3’-diaminobenzidine tetrahydrochloride in 0.01% H_2_O_2_ for 10 min. The slides were counterstained with Meyer’s hematoxylin. The negative controls were processed in a similar manner with PBS instead of primary antibody. The positive GRB2, GAB1 and p-ERK1 expression confirmed by western blotting were used as positive controls for immunostaining.

Following a hematoxylin counterstaining, immunostaining was scored by two independent experienced pathologists, who were blinded to the clinicopathological parameters and clinical outcomes of the patients. The scores of the two pathologists were compared and any discrepant scores were trained through re-examining the stainings by both pathologists to achieve a consensus score. The numbers of positive-staining cells showing immunoreactivity in the cytoplasm for GAB1 and p-ERK1, and in the cell nucleus and cytoplasm for GRB2 in ten representative microscopic fields were counted and the percentage of positive cells was calculated. The percentage scoring of immunoreactive tumor cells was as follows: 0 (0%), 1 (1-10%), 2 (11-50%) and 3 (>50%). The staining intensity was visually scored and stratified as follows: 0 (negative), 1 (weak), 2 (moderate) and 3 (strong). A final immunoreactive score (IRS) was obtained for each case by multiplying the percentage and the intensity score. Therefore, tumors with a multiplied score exceeding median of total scores for GRB2, GAB1 and p-ERK1 were deemed to be high expression groups; all other scores were considered to be low expression groups.

#### Statistical analysis

The software of SPSS version13.0 for Windows (SPSS Inc, IL, USA) and SAS 9.1 (SAS Institute, Cary, NC) was used for statistical analysis. The chi-squared test was used to show differences in categorical variables. Patient survival and the differences in patient survival were determined by the Kaplan-Meier method and the log-rank test, respectively. A Cox regression analysis (proportional hazard model) was performed for the multivariate analyses of prognostic factors. Differences were considered statistically significant when P was less than 0.05.

## Results

### Identification of candidate HCC markers

**Figure 2:**
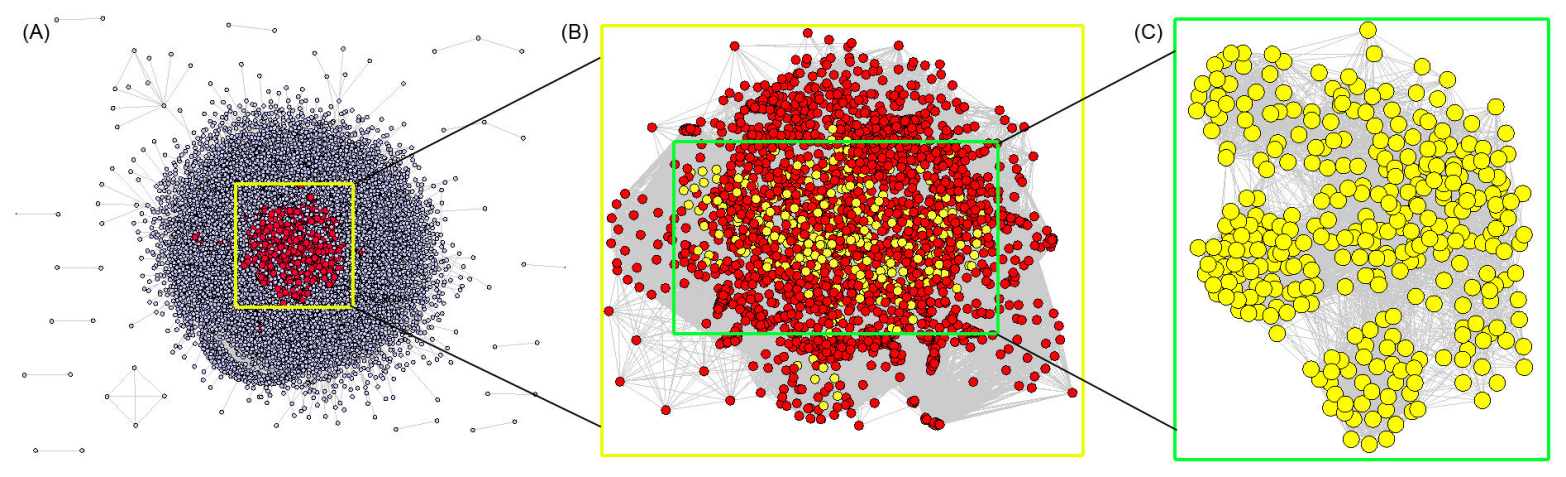
Protein-protein interaction (PPI) networks of HCC significant proteins. (A) PPI network of HCC significant proteins. This network consists of 14713 nodes and 144925 edges. (B) PPI network of hub HCC significant proteins which extracted from (A). This network consists of 14713 nodes and 61143 edges. (C) PPI network of candidate HCC markers which extracted from (B). This network consists of 331 nodes and 7207 edges.

In total, 6179 HCC significant genes and 977 HCC significant proteins which were differentially expressed or had genetic variations in HCC tissues relative to their corresponding normal tissues were collected from five existing HCC related databases (OncoDB.HCC, HCC.net, dbHCCvar, EHCO and Liverome) after removing redundancy. The detailed information on these HCC significant genes and proteins is described in Table S1. Since multiple biological processes or pathways are implicated in tumorigenesis and tumor progression of HCC, we constructed HCC related network using PPI information of HCC significant proteins. This network consists of 14713 nodes and 144925 edges (Figure 2A). According to the previous study of Li et al. [34], we identified a node as a hub protein if its degree is more than 2 fold of the median degree of all nodes in a network. The network of hub HCC significant proteins consists of 14713 nodes and 61143 edges (Figure 2B). Four topological features, 'Degree,' 'Betweenness', 'Closeness' and 'K value' (defined in 'Materials and methods' section), were chosen to identify candidate HCC markers. After calculating the value of the four features for each hub HCC significant protein in the PPI network, the median values of 'Degree', 'Betweenness', 'Closeness' and 'K value' were 67, 0.03, 39.37 and 31, respectively. Therefore, we determined that HCC significant proteins with 'Degree'>67, 'Betweenness'>0.03, 'Closeness'>39.37, and 'K value'>31 were candidate HCC markers for tumor therapy. As a result, 331 proteins were identified as candidate HCC markers. Please see detail information on topological features and the PPI network of these candidate HCC markers in Table S3 and Figure 2C, respectively.

### Enrichment analysis of candidate HCC markers

**Figure 3:**
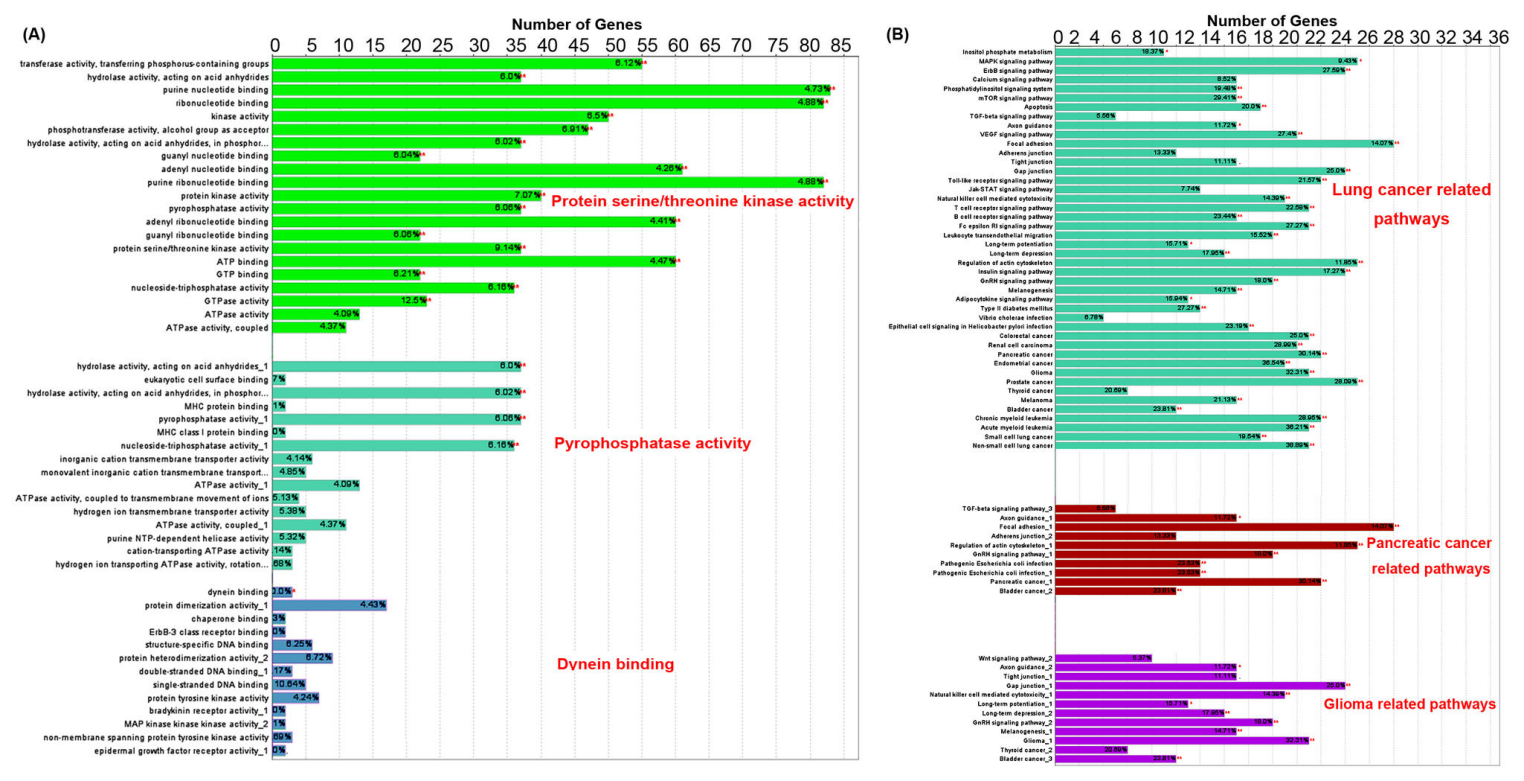
Top three significantly enriched gene ontology (GO) molecular functions (A) and KEGG pathways (B) involved by candidate hepatocellular carcinoma (HCC) markers. The functional distribution of candidate HCC markers was obtained from GO enrichment analysis. Candidate HCC markers more frequently had protein serine/threonine kinase and pyrophosphatase activities, and also were involved in dynein binding process. Since the pathway information is important for understanding gene and protein functions, we also analyzed the enriched KEGG biological pathways among these candidate HCC markers, which were most commonly implicated in cancer related pathways, such as non-small cell lung cancer, pancreatic cancer and glioma. '*'P<0.05,'**'P<0.01.

In order to investigate the biological characteristics of 331 candidate HCC markers, enrichment analysis based on GO annotation system was performed. This system uses a controlled and hierarchical vocabulary to assign function to genes or gene products in any organism. The results showed that the molecular functions of candidate HCC markers could be divided into 26 groups. Please see all the results of GO enrichment analysis on candidate HCC targets in Table S4. Figure 3A shows the top three groups of molecular functions that have the most candidate HCC markers. One of the most important molecular functions is protein serine/threonine kinase activity. There were 99 candidate HCC markers with this activity. For example, the cyclin-dependent kinases (CDKs), key regulators of the cell cycle, have been demonstrated to be activated in HCC and promote the tumor progression [35]; the mitogen-activated protein kinases (MAPKs), as a focal point for signal transduction following activation of both G-protein-linked and tyrosine kinase growth factor receptors, have been reported to be involved in the regulation of apoptosis of tumor cells and be associated with aggressive progression of HCC [36]. In the biological process category, the candidate HCC markers more frequently play roles in cellular protein metabolic process, translational elongation and intracellular signaling cascade, which are associated with cancer development and metastasis of HCC. 

In terms of pathway information which is important for understanding gene and protein function, cancer related pathways, such as MAPK signaling pathway, mTOR signaling pathway, and focal adhesion, were the most associated pathways of candidate HCC markers (Figure 3B). Please see all the results of KEGG pathway enrichment analysis in Table S4.

### Identification of crucial candidate HCC markers for experimental validation

**Figure 4:**
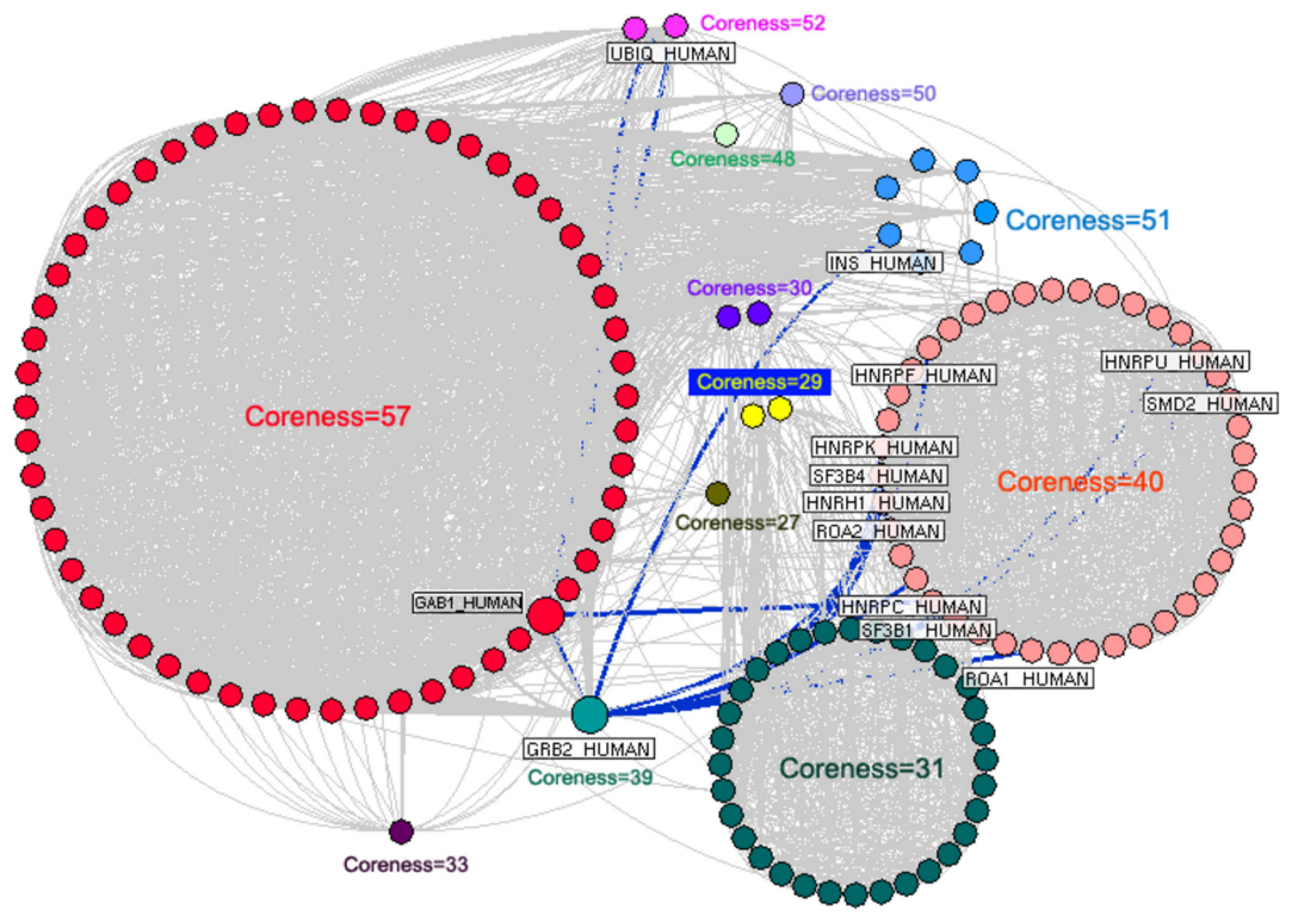
Co-expression network of 331 candidate HCC markers constructed using the K-core analysis. Nodes in different cores are marked with various colors. Blue edges refer to the shortest paths through the interaction between GRB2 and GAB1 for the connection of different cores.

In order to identify the crucial candidate HCC markers for experimental validation from 331 candidate HCC markers, a co-expression network was constructed using the K-core analysis. The k values of candidate HCC markers were ranged from 9 to 57. Please see detail information on k values of candidate HCC markers in Table S3. We showed the 12 innermost cores of the co-expression network in Figure 4. Fifty-eight candidate HCC markers, such as eukaryotic translation initiation factor 3 subunit E (EIF3E), GAB1, and eukaryotic translation initiation factor 5 (EIF5), etc. were located in the innermost core (k=57).

Furthermore, we calculated the edge-betweenness to find the most important PPI in the co-expression network. As the result (Please see detail information on edge-betweenness of the co-expression network in Table S5), the interaction between GRB2 and GAB1 had the highest edge-betweenness and connected different cores in the co-expression network (Figure 4). 

Since GAB1 has the highest k-coreness suggesting its central localization in HCC related network, and the interaction between GRB2 and GAB1 has the largest edge-betweenness implying it may be biologically important to the function of HCC related network, we performed the experimental validation to evaluate the clinical significance of GRB2 and GAB1 in HCC.

### Experimental Validation

#### Upregulation of GRB2 and GAB1 proteins in HCC tissues

**Figure 5:**
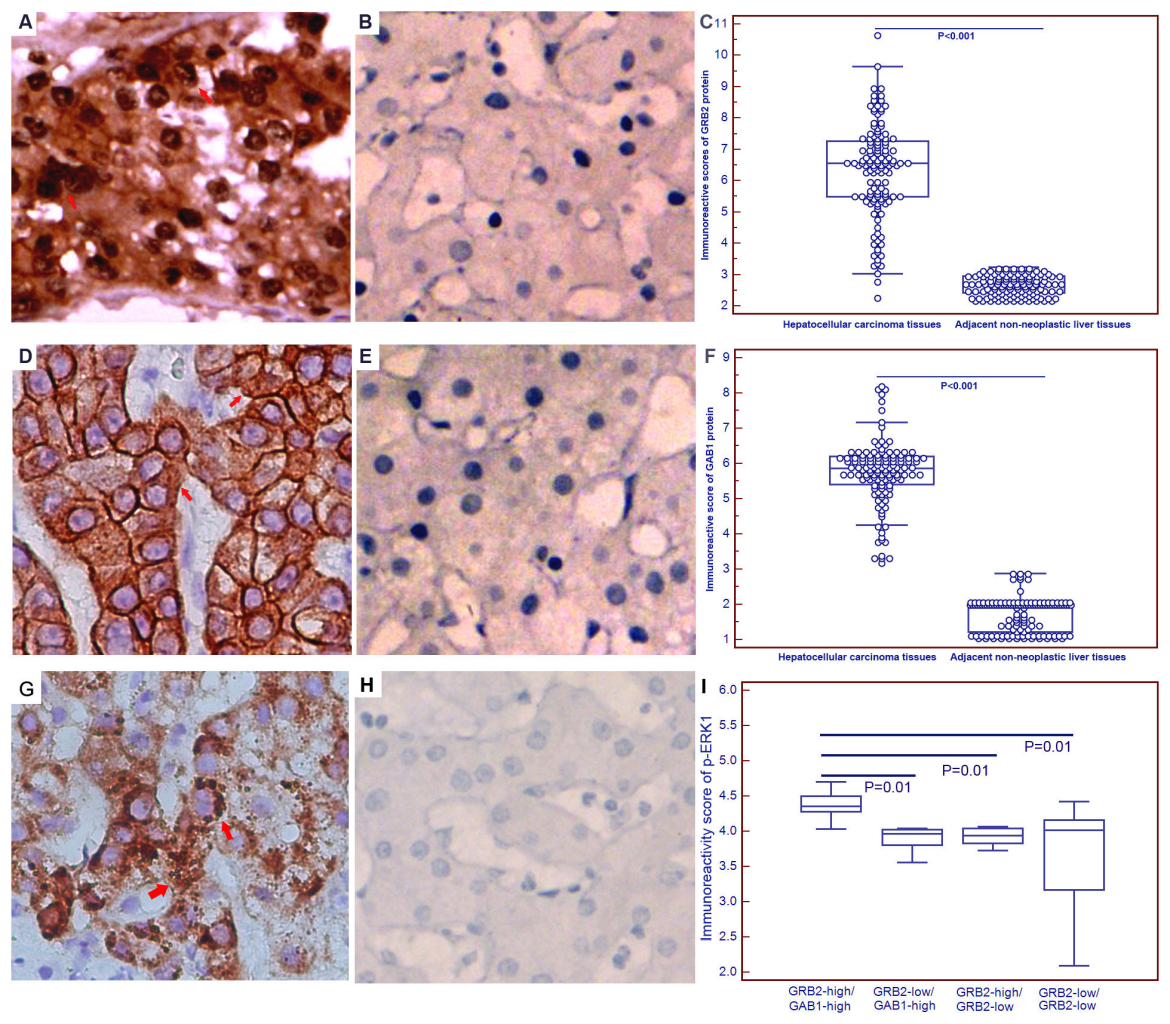
Representative immunohistochemical images of GRB2 (A and B), GAB1 (D and E) and p-ERK1 (G and H) expression in HCC and adjacent non-neoplastic liver tissues (Original magnification×400). Statistical analyses of IRS for GRB2 (C), GAB1 (F) and p-ERK1 (I) immunostainings in HCC and adjacent non-neoplastic liver tissues. GRB2 positive staining was localized in the cell nucleus and cytoplasm, while GAB1 positive staining was localized in the cytoplasm of tumor cells in HCC tissues. Compared with the adjacent nonneoplastic tissues, the expression levels of GRB2 (IRS for HCC vs. nonneoplastic liver: 6.32±1.50 vs. 2.67±0.32, P<0.001) and GAB1 (IRS for HCC vs. nonneoplastic liver: 5.72±0.95 vs. 1.75±0.48, P<0.001) proteins were all significantly increased in HCC tissues. More interestingly, in all four groups according to the combined expression of GRB2 and GAB1, GRB2-high/GAB1-high patients expressed the highest level of p-ERK1 protein (all P=0.01).

The subcellular localization and the expression pattern of GRB2 and GAB1 proteins in 130 self pairs of HCC and adjacent nonneoplastic liver tissues were observed by the immunohistochemistry analysis. As shown in Figure 5A and 5D, GRB2 positive staining was localized in the cell nucleus and cytoplasm, while GAB1 positive staining was localized in the cytoplasm of tumor cells in HCC tissues. Compared with the adjacent nonneoplastic tissues, the expression levels of GRB2 (IRS for HCC vs. nonneoplastic liver: 6.32±1.50 vs. 2.67±0.32, P<0.001, Figure 5C) and GAB1 (IRS for HCC vs. nonneoplastic liver: 5.72±0.95 vs. 1.75±0.48, P<0.001, Figure 5F) proteins were all significantly increased in HCC tissues. Based on the scoring system used in the present study, 56 (43.08%) cases were both high expression of GRB2 and GAB1, 54 (41.54%) cases were both low expression of GRB2 and GAB1, 9 (6.92%) cases were GRB2-high and GAB1-low expression, and 11 (8.46%) cases were GRB2-low and GAB1-high expression. As determined by Spearman’s correlation, the GRB2 expression was significantly associated with the GAB1 expression (r=0.68, P=0.01).

In order to validate the hypothesis that GRB2 and GAB1 can induce the activation of the HGF/MAPK/ERK pathway, we detected the expression of p-ERK1 protein in 130 self pairs of HCC and adjacent nonneoplastic liver tissues. As shown in Figure 5G, p-ERK1 positive staining was localized in the cytoplasm of tumor cells in HCC tissues. Compared with the adjacent nonneoplastic tissues, the expression level of p-ERK1 (IRS for HCC vs. nonneoplastic liver: 4.04±0.67 vs. 1.20±0.33, P<0.001) protein was significantly increased in HCC tissues. More interestingly, in all four groups according to the combined expression of GRB2 and GAB1, GRB2-high/GAB1-high patients expressed the highest level of p-ERK1 protein (all P=0.01, Figure 5I).

#### Upregulation of GRB2 and GAB1 proteins associates with the aggressive tumor progression of HCC

To evaluate whether GRB2 and GAB1 protein expression was associated with clinicopathological features of patients with HCC, we correlated IRS of GRB2 and GAB1 proteins with tumor stage, tumor grade, serum AFP level, presence of cirrhosis, underlying liver disease including alcohol abuse, viral hepatitis B and C, sex, and age (Table 1). As the results, we found that the expression levels of GRB2 protein in HCC tissues with the higher tumor stage (T3~4) and the positive serum AFP level were significantly lower than those with the lower tumor stage (T1~2, P=0.01, Table 1) and the negative serum AFP level (P=0.006, Table 1), respectively. In addition, the frequencies of aberrant GAB1 expression were higher in HCC tissues with higher tumor stage (T3~4) than those with lower tumor stage (P=0.01, Table 1). GAB1 overexpression was also observed more frequently in HCC tissues with high tumor grade than those with low grade (P=0.02, Table 1). More importantly, the combined GRB2 and GAB1 protein expression was significantly associated with serum AFP (P=0.01, Table 1), tumor stage (P=0.006, Table 1) and tumor grade (P=0.02, Table 1).

#### Upregulation of GRB2 and GAB1 proteins predicts the poor prognosis in patients with HCC

**Figure 6:**
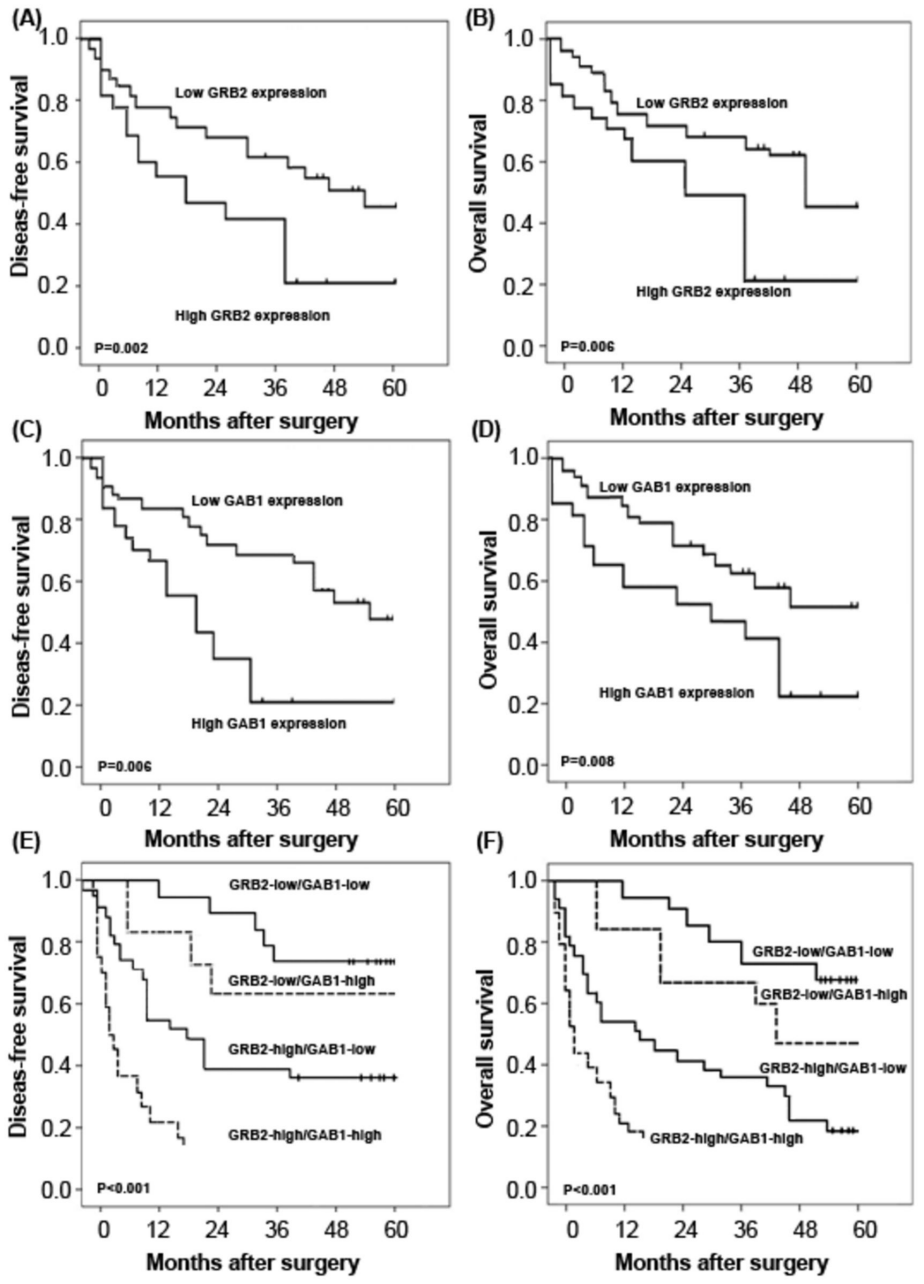
Disease-free survival and overall survival curves for two groups defined by low and high expression of GRB2 (A and B) and GAB1 (C and D), and for four groups defined by combined expression of GRB2 and GAB1 (E and F), in patients with HCC. The patients with high GRB2 and GAB1 expression had a significantly shorter 5-year overall and disease-free survival rate than those with low GRB2 and GAB1 expression (both P=0.008). In addition, the results by pairwise comparisons showed that the statistically significant difference of overall and disease-free survival existed between GRB2-high/GAB1-high patients and any of other three groups (both P<0.001). In all four groups, GRB2-high/GAB1-high patients had the poorest prognosis.

Five-year disease-free survival was observed in 30 (23.08%) patients, whereas in 100 (76.92%) patients, disease recurred, and 88 (67.69%) even died during a 5-year follow-up period. We observed a trend that 5-year disease-free survival in the group with high GRB2 expression was significantly poorer than that in the group with low GRB2 expression (P=0.002, log-rank test; Figure 6A). Additionally, the Kaplan-Meier plot of 5-year overall survival curves stratified by GRB2 expression was shown in Figure 6B. A significant relationship was found between GRB2 expression and 5-year overall survival (P=0.006, log-rank test, Figure 6B). Similar with GRB2, the disease-free survival (Figure 6C, P=0.006) and overall survival (Figure 6D, P=0.008) of HCC patients with high GAB1 expression were both significantly shorter than those with low GAB1 expression. Moreover, the association between the co-expression of GRB2/GAB1 and the survival rates were tested by the method of Kaplan-Meier. The Chi-square value by Log Rank (Mantel-Cox) indicated a significant difference among different groups with regard to the conjoined expression status of GRB2/GAB1 (Figure 6E and F). The results by pairwise comparisons showed that the statistically significant difference of disease-free survival and overall survival existed between GRB2-high/GAB1-high patients and any of other three groups (both P<0.001). In all four groups, GRB2-high/GAB1-high patients had the poorest prognosis.

Furthermore, in a multivariate Cox model, including serum AFP, tumor stage, tumor grading, presence of cirrhosis, gender, age, GRB2 expression, GAB1 expression and combined GRB2/GAB1 expression, we found that GRB2 expression (both P=0.01, Table 2), GAB1 expression (both P=0.01, Table 2) and combined GRB2/GAB1 expression (both P=0.001, Table 2) were independent poor prognostic factors for both 5-year disease-free survival and 5-year overall survival in HCC.

**Table 2:** Multivariate survival analysis of five-year overall and disease-free survival in 130 patients with hepatocellular carcinoma.

**Features**	**Five-year overall survival**			**Five-year disease-free survival**		
	**HR**	**95% CI**	**P**	**HR**	**95% CI**	**P**
Age	1.132	0.316-3.516	0.192	1.536	0.322-3.736	0.125
Gender	1.191	0.345-3.857	0.136	1.559	0.357-3.831	0.131
Serum AFP	1.931	0.685-4.056	0.063	1.953	0.615-4.273	0.062
Tumor stage	2.879	1.366-5.196	0.009	2.686	1.386-6.009	0.01
Tumor grade	1. 563	0.609-4.088	0.081	1.551	0.607-4.466	0.086
Presence of cirrhosis	1.919	0.738-4.102	0.063	1.921	0.793-4.219	0.062
GRB2 expression	3.798	1.205-8.038	0.01	3.820	1.213-8.186	0.01
GAB1 expression	3.683	1.166-7.679	0.01	3.892	1.172-7.889	0.01
GRB2/GAB1 co-expression	5.829	1.309-12.061	0.001	5.951	1.318-12.226	0.001

## Discussion

Since crucial molecules often show relatively slight changes between groups of samples (e.g. normal vs. disease), identifying disease related molecules only from differential analysis of high-throughput data may be poorly annotated and lack of biological significance. Our previous studies suggest that organized knowledge, such as molecular interaction networks and biological pathways, may help high-throughput data analysis in significant ways [11,12]. In the current study, we combined the expression profile and interaction network analyses to identify a list of biologically significant HCC related markers and pathways. We also validated the clinical significance of crucial candidate HCC markers for human HCC.

Based on network theory, cancer genes and proteins do not function in isolation; instead, they work in interconnected pathways and molecular networks at multiple levels [37]. Here, we constructed the PPI network of HCC significant proteins obtained from high-throughput detection and screened the candidate HCC markers by measuring the topological characteristics. We calculated degree centrality, betweenness centrality and closeness centrality of a node, and also evaluated the importance of co-expression sub-network by K-core analysis and the influence of PPI in information transfer by edge-betweenness algorithm. In large real world networks, the node degree distributions often show a heavy tail, which means that there are a few, but not zero, nodes with very high node degrees. These nodes are frequently called hubs, and play a critical role in the network [38]. On this basis, we identified 2098 hub HCC significant proteins from differential expression profile. In addition, we calculated degree centrality, betweenness centrality, closeness centrality and K-coreness to identify 'Central' nodes which are important and can reach the whole network more quickly than non-central nodes as candidate HCC markers. Moreover, the K-core analysis was performed to construct k-core sub-networks of candidate HCC markers in order to uncover the co-expression modules which are embedded in the center of the network. Our data found that GAB1 was located in the innermost core. More importantly, the results of the edge-betweenness algorithm showed that the interaction between two candidate HCC markers--GRB2 and GAB1 had the highest edge-betweenness, suggesting that there may be the largest number of shortest paths through this interaction which may play a crucial role in connecting various sub-networks in HCC related network.

Furthermore, we determined the expression patterns of GRB2 and GAB1 proteins in 130 HCC tissues and paired adjacent non-neoplastic tissues using immunohistochemistry analysis. GRB2 is a ubiquitously expressed adapter protein composed of one SH2 domain flanked by amino- and carboxy-terminal SH3 domains [39]. It provides a critical link between cell surface growth factor receptors and the Ras signaling pathway [40]. Accumulating studies have demonstrated the importance of GRB2 in the oncogenesis of several important human malignancies. Functionally, GRB2 contributes to tumor growth, invasiveness and metastasis making it a high priority target for anti-cancer drug development [41]. GAB1 belongs to the Gab family which has emerged as crucial signaling compartments in metazoans [42]. GAB1 is involved in the amplification and integration of signal transduction evoked by a variety of extracellular stimuli, including growth factors, cytokines, antigens, and other molecules [43]. GAB1 plays a role in tumorigenesis by involving in c-Met receptor signaling, since c-Met is activated, mutated, or overexpressed in a wide range of cancers [44]. It also functions as a mediator of EGFR-signaling-induced tumorigenesis in glioblastomas and intestinal adenomas [45]. Notably, the interaction between GRB2 and GAB1 mediates signaling between upstream cell surface receptor tyrosine kinases (RTKs) and downstream effectors such as Ras and Akt involved in a diverse array of cellular activities including growth, survival, proliferation and oncogenic transformation [46]. The disruption of this interaction may be implicated in oncogenesis of various human cancers. In HCC, Yoon et al. [47] indicated that the GRB2-mediated signaling pathway may be involved in tumor progression and differentiation of hepatocarcinoma cells; Kondo et al. [48] reported that coupling of GRB2 to GAB1 could mediate the HGF-induced strong activation of the ERK pathway, which is required for the inhibition of HepG2 cell proliferation. In the current study, we confirmed that the overexpression of GRB2 mainly occurred in the cell nucleus and cytoplasm in HCC tissues relative to adjacent non-neoplastic tissues, and that GAB1 expression was markedly upregulated in the cytoplasm of tumor cells in HCC tissues compared with paired adjacent non-neoplastic tissues. In addition, our data showed that the HCC tissues showing high expression of both GRB2 and GAB1 also displayed the activation of ERK1 protein, suggesting that GRB2 and GAB1 might induce the activation of the HGF/MAPK/ERK pathway, which has been tied to oncogenic transformation and cancer progression in HCC [49,50]. Moreover, both the increased expression of GRB2 and GAB1 proteins were significantly associated with aggressive clinicopathological features of HCC. Interestingly, the coexpression of GRB2 and GAB1 may be associated with serum AFP, tumor stage, tumor grade and patient prognosis. To the best of our knowledge, this is the first study to identify the coexpression of GRB2 and GAB1 as a useful diagnostic and prognostic marker for HCC patients.

In conclusion, this study provided an integrative analysis by combining expression profile and interaction network analysis to identify a list of biologically significant HCC related markers and pathways. Further experimental validation indicated that the aberrant expression of GRB2 and GAB1 proteins may be strongly related to tumor progression and prognosis in patients with HCC. The overexpression of GRB2 in combination with upregulation of GAB1 may be an unfavorable prognostic factor for HCC. However, large scale studies will be required for further verification of the critical roles of other candidate HCC markers in the development and progression of HCC.

## Supporting Information

Table S1 **Detailed information on these hepatocellular carcinoma (HCC) significant genes and proteins.**
(XLSX)

Table S2 **Detailed information on eight existing protein-protein interaction databases.**
(XLSX)

Table S3 **Topological features of 331 candidate HCC markers.**
(XLSX)

Table S4 **Enrichment analysis of candidate hepatocellular carcinoma (HCC) markers on gene ontology (GO) molecular functions and biological processes, and KEGG pathways.**
(XLSX)

Table S5 **Edge-betweenness of the interactions in the co-expression network of 331 candidate HCC markers.**
(XLSX)
